# Is Age Just a Number? A Comparative Analysis of Glomerular Disease Across Ages from the Cure Glomerulonephropathy Network

**DOI:** 10.34067/KID.0000001090

**Published:** 2025-12-23

**Authors:** Andrew Vissing, Joseph Fishbein, Abigail R. Smith, Shikha Wadhwani, Jerome C. Lane, Jill Krissberg

**Affiliations:** 1 Division of Nephrology and Hypertension, Northwestern University Feinberg School of Medicine, Chicago, Illinois; 2 Division of Nephrology, Ann and Robert H. Lurie Children's Hospital of Chicago, Chicago, Illinois; 3 Division of Biostatistics, Department of Preventative Medicine, Northwestern University Feinberg School of Medicine, Chicago, Illinois; 4 Division of Nephrology, University of Texas Medical Branch, Galveston, Texas

**Keywords:** chronic GN, clinical nephrology, epidemiology and outcomes, glomerular disease, IgA nephropathy, pediatric nephrology, FSGS, minimal change disease

## Abstract

**Key Points:**

- Adolescents and young adults with glomerular disease have unique clinical features distinct from children and older adults.
- Patterns of relapse, remission, and changes in kidney function differ by age and glomerular disease type.
- Traditional pediatric-adult cutoffs often overlook key differences; care and research should reflect a continuum across the lifespan.

**Background:**

Glomerular disease (GD) is a prominent cause of kidney disease in adolescents and young adults (AYAs), yet there is limited information on how this population fares compared with children and older adults.

**Methods:**

We analyzed data from Cure Glomerulonephropathy, a prospective cohort of patients of all ages with biopsy-proven GD. Patients with minimal change disease (MCD), FSGS, and IgA nephropathy (IgAN) were included. Patients were stratified into pediatric (≤13), AYA (14–25), and adult (≥26) groups and compared by demographic, clinical, and disease characteristics. Associations between age group and relapse rate, change in kidney function, and time to remission were assessed using multivariate negative binomial, linear mixed effects, and Cox proportional hazards models, respectively, stratified by disease type.

**Results:**

Our study included 1868 patients (562 pediatric, 397 AYA, and 909 adults). The median follow-up time was 4.9 years. Adults with MCD had fewer relapses (incidence rate ratio [IRR], 0.61; 95% confidence interval [CI], 0.41 to 0.91; *P* = 0.01), while there was no difference between pediatric participants with MCD (IRR, 1.23; 95% CI, 0.85 to 1.79; *P* = 0.28) compared with AYA. Adults with IgAN had fewer relapses than AYA (IRR, 0.55; 95% CI, 0.33 to 0.94; *P* = 0.03). AYA had faster decline in kidney function compared with pediatric participants with FSGS (1.7 versus 0.3 ml/min per 1.73 m^2^ per year, *P* = 0.008) and IgAN (1.5 versus 0.1 ml/min per 1.73 m^2^ increase per year, *P* = 0.002). Pediatric participants with MCD achieved first observed remission sooner compared with AYA (hazard ratio, 2.18; 95% CI, 1.03 to 4.63; *P* = 0.04). Adults with IgAN were slower to achieve first observed remission compared with AYA (hazard ratio, 0.58; 95% CI, 0.37 to 0.91; *P* = 0.02).

**Conclusions:**

AYA with GD exhibit distinct clinical patterns compared with the pediatric and adult age groups, underscoring the need to approach care and research along an age-related continuum rather than a binary framework.

## Visual Abstract

**Figure:**
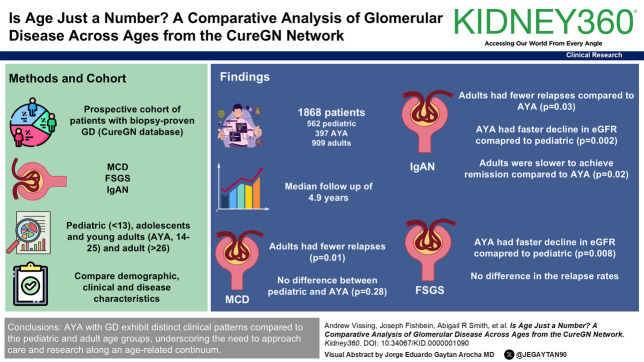

## Introduction

The population of adolescents and young adults (AYAs) living with kidney disease is growing.^1^ The AYA period, spanning from mid-teens through mid-twenties, is marked by more independence from guardians, increasing personal responsibility and exposure to new psychosocial stressors. In addition to these changes, this group faces unique challenges when it comes to living with their chronic condition that impact their health outcomes. Risk-taking behaviors, medication nonadherence, and difficulty navigating health care systems are more prevalent in this population, all of which complicate management of their disease.^2^ AYA with kidney disease also experience cognitive deficits, lower self-esteem, limited social support, and barriers to education and employment further contributing to poor health outcomes.^3–5^

In addition to these developmental and psychosocial hurdles, AYA must navigate the health care transition from pediatric to adult health care—a process associated with care fragmentation, decreased engagement, and increased acute care utilization.^6^ For AYA with kidney transplants, this has translated to higher rates of acute graft rejection and graft loss.^7–12^ Glomerular disease (GD) is a leading cause of ESKD in the AYA population, accounting for over 20% of cases; yet despite its significance, limited data exist specifically on outcomes for AYA with GD.^13^

Most studies examining outcomes in GD use a conventional pediatric-adult division—often arbitrarily set at age 18 years. However, this approach does not accurately reflect the biologic and psychosocial continuum of the AYA experience.^14–17^ A growing body of literature on GD has begun considering adolescents and/or young adults as a distinct clinical group; however, there is no consensus for age cutoffs, making it difficult to draw generalizations for this demographic.^1,18–21^

Our study aims to better characterize the AYA population with GD and to compare their disease characteristics and outcomes with those of children and older adults. By doing so, we hope to help inform guidelines and treatment practices for this vulnerable group. We hypothesized that AYA with GD would have worse clinical trajectories than pediatric and older adult patients, with higher relapse rates, faster decline in kidney function, and slower remission. This expectation was based on prior evidence of studies of AYA with kidney transplants as well as the developmental, psychosocial, and health care transition challenges unique to the AYA period.

## Methods

The Cure Glomerulonephropathy (CureGN) Network is an ongoing, multicenter, multinational prospective cohort study of children and adults of all ages with biopsy-proven GD. Participants enroll within 5 years of diagnostic kidney biopsy. CureGN includes patients with IgA nephropathy (IgAN; including IgA vasculitis), minimal change disease (MCD), FSGS, and membranous nephropathy (MN). Enrollment data collection includes demographic and baseline disease characteristics. Laboratory data, medications, and symptoms are collected at subsequent visits.^22^ This study was deemed exempt by the Northwestern University Institutional Review Board as it does not involve human subjects research.

We included patients of all ages who completed an enrollment visit and had at least 12 months of follow-up in this study. We excluded patients with MN given its low frequency in the pediatric age group, which made meaningful comparative analysis difficult for this entity. The primary exposure of interest was age at enrollment into CureGN. Patients were categorized into three age groups: pediatric (13 years of age and younger), AYA (14–25 years of age), and adult (26 years and older).

Kidney function was calculated using the 2021 CKD Epidemiology Collaboration equation for patients 25 years and older, and the CKD in Children Study under 25 equation was used for patients younger than 25 years.^23,24^ Values over 140 ml/min per 1.73 m^2^ were winsorized at 140 ml/min per 1.73 m^2^ to account for potential hyperfiltration. Active disease at enrollment was defined by CureGN and varied by disease subtype (Table 1). For patients 18 years and older, hypertension was defined as a systolic BP (SBP) >130 mm Hg and diastolic BP (DBP) >80 mm Hg. For patients younger than 18 years, hypertension was defined as SBP or DBP greater than the 95th percentile for age, sex and height, or a SBP >130 mm Hg or DBP >80 mm Hg.

**Table 1:** Outcome definitions by glomerular disease subtype

Definition	Disease	Requirements (Any One of the Following)
Active disease at the time of enrollment	MCD	UPCR >1 mg/mg
		Dipstick protein 1+ or more
		Reported pattern of frequently relapsing or steroid dependent nephrotic syndrome^a^
		One or more relapses within 12 mo of enrollment
		Use of immunosuppressive mediations at enrollment
	FSGS	UPCR >1 mg/mg
		24-h urine protein >1 g/d
	IgAN	UPCR >0.3 mg/mg
		24-h urine protein >500 mg/d
		Gross hematuria
		Dipstick blood 1+ or more
Relapse	MCD, FSGS, IgAN	UPCR >3 mg/mg
		24-h urine protein >3 g/d
		Dipstick protein 3+ or more
		Reported relapse by study investigators
	IgAN only	Gross hematuria
		Dipstick blood 3+ or more
Remission	MCD, FSGS, IgAN	UPCR <0.3 mg/mg
		24-h urine protein <300 mg/d
		Dipstick protein negative or trace for at least 3 consecutive days
		Reported remission by study investigators
	IgAN only	Absence of hematuria (urine dipstick blood negative or trace) and normal urine protein as defined as one of the above

IgAN, IgA nephropathy; MCD, minimal change disease; UPCR, urine protein-to-creatinine ratio.

a Frequently relapsing or steroid dependent nephrotic syndrome was determined by the treating physician. There was no study-wide definition.

Our primary outcome of interest was relapse rate which was calculated as the number of relapses per 100 patient-years starting at the time of enrollment. To be included in analysis, the individual needed to be considered at-risk for relapse, *i.e*., individuals who had achieved complete remission before enrollment, at the time of enrollment, or at some point during the follow-up period. Relapse rates were stratified by age group and disease subtype. Clinical criteria for relapse and remission for this primary outcome are presented in Table 1.

Secondary outcomes included change in eGFR and time from enrollment or first relapse (if in remission at enrollment) to first observed remission. Participants not meeting the definition of remission at enrollment were followed until the earliest onset of remission, last study follow-up visit, or 5 years from enrollment. Those in remission at enrollment were included if they were observed to leave the remission state at some point during follow-up, defined as evidence of urine protein-to-creatinine ratio (UPCR) >1.0 g/g or urine dipstick of 2+ or greater. They were then followed until the earliest onset of remission, last study follow-up visit, or 5 years from enrollment. Participants with no evidence of leaving the defined remission state were excluded from these models.

### Statistical Analysis

Demographic and baseline disease characteristics collected at enrollment were compared across age groups. Nonparametric tests were used to compare medians, and chi-squared tests were used to compare categorical variables. Relapse rates were compared using incidence rate ratios (IRRs) calculated from negative binomial models with the AYA group as the reference. Change in eGFR was analyzed using linear mixed effects regression models with random intercepts and slopes on all eGFR values available in the first 5 years after enrollment. Difference in rate of change of eGFR was assessed using interactions between age group and years from enrollment, with AYA as the reference, as well as within disease subtypes. Time to first observed remission during prospective CureGN follow-up was analyzed using multivariable Cox regression. All models were adjusted for sex, time from diagnosis to enrollment, UPCR, hypertension status, body mass index, and insurance type. All analyses were completed using SAS Enterprise Guide, version 7.15 (SAS Institute, Cary, NC).

## Results

Of the 1868 patients included in the study, 562 (30%) were pediatric, 397 (21%) were AYA, and 909 (49%) were adults (Table 2). The median follow-up time was 4.9 years. The median disease duration before enrollment was 1.5 years for pediatric participants, 1.3 years for AYA, and 1.3 years for adults. The prevalence of GD in the study varied by age with MCD being the most common GD in pediatric participants (50%) and IgAN most common in AYA (44%) and in adults (42%). More pediatric participants had active disease at enrollment (79% versus 69% in AYA and 69% in adults) but were also more likely to have reached complete remission at least once before enrollment (51% versus 34% in AYA and 24% in adults). Adults exhibited the lowest eGFR at enrollment (64 versus 89 in AYA and 103 in pediatric participants, measured in ml/min per 1.73 m^2^). Adults had the highest UPCR at enrollment (1.1 versus 0.6 mg/mg in AYA and 0.3 mg/mg in pediatric participants). Adults had higher prevalence of hypertension at enrollment (63%) compared with AYA (36%) and pediatric participants (34%).

**Table 2:** Demographic and baseline characteristics

Charecteristic	Pediatrics (*n*=562)	AYA (*n*=397)	Adults (*n*=909)
Age at enrollment (yr)	8 (5–11)	17 (15–20)	45 (35–58)
Age at biopsy (yr)	6 (4–9)	15 (14–18)	43 (33–55)
Disease duration (yr)	1.5 (0.5–3.4)	1.3 (0.4–3.1)	1.3 (0.5–3.4)
Time from biopsy to enrollment (mo)	11 (3–29)	13 (3–32)	14 (5–36)
Active disease at enrollment	441 (79%)	275 (69%)	623 (69%)
Complete remission before enrollment	285 (51%)	133 (34%)	213 (24%)
Male	332 (59%)	229 (58%)	484 (53%)
**Race**			
Asian	38 (7%)	17 (4%)	106 (12%)
Black	78 (14%)	79 (20%)	118 (13%)
Other	38 (7%)	13 (3%)	29 (3%)
Unknown	17 (3%)	11 (3%)	42 (5%)
White	391 (70%)	277 (70%)	614 (68%)
**Ethnicity**			
Hispanic	68 (12%)	53 (13%)	135 (15%)
Non-Hispanic	493 (88%)	343 (86%)	774 (85%)
**Disease**			
MCD	280 (50%)	93 (23%)	183 (20%)
FSGS	130 (23%)	131 (33%)	346 (38%)
IgAN	152 (27%)	173 (44%)	380 (42%)
**Insurance type**			
Private	241 (43%)	246 (62%)	631 (69%)
Public	293 (52%)	128 (32%)	169 (19%)
Other	25 (5%)	15 (4%)	86 (10%)
None	3 (1%)	8 (2%)	23 (3%)
eGFR, ml/min per 1.73 m^2^	103 (86–122)	89 (69–103)	64 (40–92)
UPCR, mg/mg	0.3 (0.1–2.8)	0.6 (0.1–2.0)	1.1 (0.4–3.1)
Serum albumin, g/dl	3.8 (2.9–4.2)	3.9 (3.3–4.3)	3.9 (3.4–4.2)
BMI, kg/m^2^	19 (17–22)	25 (21–31)	28 (25–33)
Hypertension	189 (34%)	141 (36%)	568 (63%)
Prior RAAS blockade	491 (87%)	354 (89%)	846 (93%)
Prior IST	537 (96%)	366 (92%)	808 (89%)

Results are reported as median (interquartile range) or *n* (%). AYA, adolescent and young adult; BMI, body mass index; IgAN, IgA nephropathy; IST, immunosuppressive therapy; MCD, minimal change disease, RAAS, renin-angiotensin-aldosterone system; UPCR, urine protein-to-creatinine ratio.

**Figure 1:**
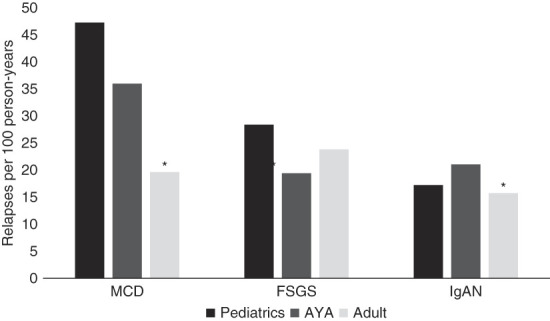
**Relapse rate by age group and GD subtype.** *Indicates a statistically significant difference when compared with AYA. AYA, adolescent and young adult; GD, glomerular disease; IgAN, IgA nephropathy; MCD, minimal change disease.

Among those with MCD, pediatric participants had 48 relapses per 100 patient-years, AYA had 36 relapses per 100 patient-years, and adults had 20 relapses per 100 patient-years. Adults experienced fewer relapses than AYA (IRR, 0.61; 95% confidence interval [CI], 0.41 to 0.91; *P* = 0.01). There was no difference observed in relapse rates between pediatric participants and AYA with MCD (IRR, 1.23; 95% CI, 0.85 to 1.79; *P* = 0.28; Figure 1). Among those with FSGS, pediatric participants had 29 relapses per 100 patient-years, AYA had 20 relapses per 100 patient-years, and adults had 24 relapses per 100 patient-years. No difference was detected in the relapse rates in FSGS for AYA versus pediatric participants or AYA versus adults. Among patients with IgAN, pediatric participants had 17 relapses per 100 patient-years, AYA had 21 relapses per 100 patient-years, and adults had 16 relapses per 100 patient-years. Adults with IgAN had fewer relapses than AYA (IRR, 0.55; 95% CI, 0.33 to 0.94; *P* = 0.03). There was no difference observed in relapse rates between pediatric participants and AYA with IgAN (IRR, 0.75; 95% CI, 0.43 to 1.29; *P* = 0.30).

**Figure 2:**
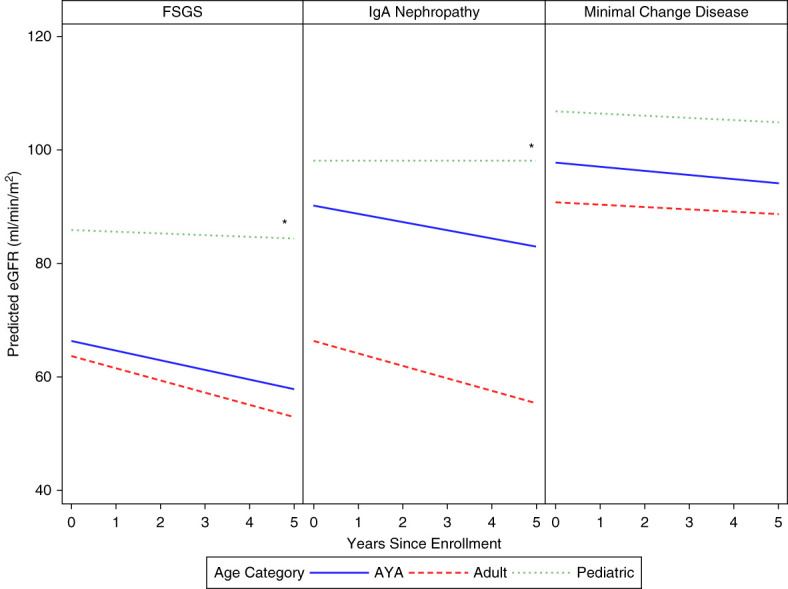
**Predicted change in eGFR by age group and GD subtype.** *Indicates a statistically significant difference when compared with AYA.

Among those with MCD, AYA had an estimated average eGFR decline of 0.7 ml/min per 1.73 m^2^ per year, pediatric participants had decline of 0.3 ml/min per 1.73 m^2^ per year, and adults had decline of 0.4 ml/min per 1.73 m^2^ per year. No significant difference was detected in the rate of eGFR change between AYA and pediatric participants or AYA and adults (Figure 2). For those with FSGS, AYA had eGFR decline of 1.7 ml/min per 1.73 m^2^ per year, pediatric participants had decline of 0.3 ml/min per 1.73 m^2^ per year, and adults had decline of 2.2 ml/min per 1.73 m^2^ per year. Age was associated with different rates of change in eGFR, with AYA demonstrating faster decline compared with pediatric participants (*P* = 0.008). No difference was detected in rate of change in eGFR between AYA and adults (*P* = 0.3). For those with IgAN, AYAs had eGFR decline of 1.5 ml/min per 1.73 m^2^ per year, pediatric participants had eGFR increase of 0.1 ml/min per 1.73 m^2^ per year, and adults had eGFR decline of 2.2 ml/min per 1.73 m^2^ per year. Age was associated with a faster rate of decline among AYA when compared with pediatric participants (*P* = 0.002), but no difference was detected between AYA when compared with adults (*P* = 0.06; Figure 2).

**Figure 3:**
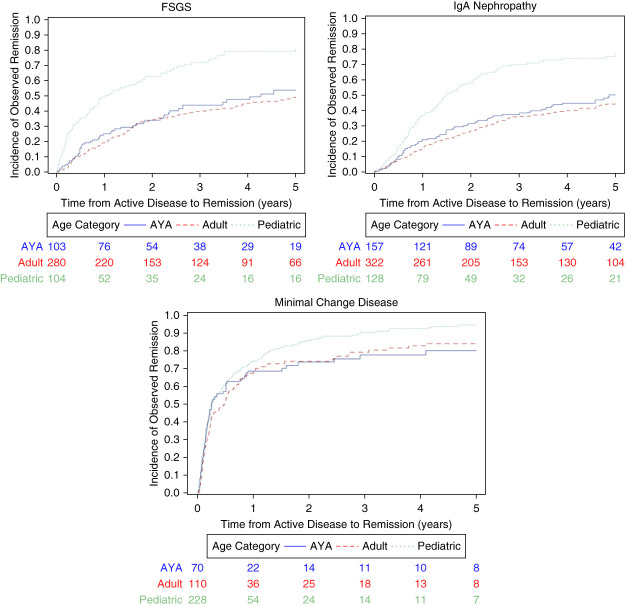
**Time to first observed remission by age group and GD subtype.** Unadjusted cumulative incidence plots comparing time from active disease or relapse to first observed remission among pediatric, AYA, and adult patients, stratified by GD subtype.

A total of 1502 patients were included in time to first observed remission analysis. Pediatric participants with MCD had a higher hazard of achieving first observed remission compared with AYA (hazard ratio [HR], 2.18; 95% CI, 1.03 to 4.63; *P* = 0.04). Pediatric participants with FSGS trended toward a higher hazard ratio for time to first observed remission compared with AYA but did not reach statistical significance (HR, 1.75; 95% CI, 0.97 to 3.18; *P* = 0.07). There was no difference observed between pediatric participants and AYA with IgAN (HR, 1.18; 95% CI, 0.68 to 2.05; *P* = 0.57; Figure 3 and Table 3). For comparison between adults and AYA, there was significant difference for IgAN only, whereas adults had a lower hazard of achieving remission compared with AYA (HR, 0.58; 95% CI, 0.37 to 0.91; *P* = 0.02). No difference was detected in time of achieving remission between adults and AYA with MCD (HR, 1.75; 95% CI, 0.77 to 3.98; *P* = 0.18) or FSGS (HR, 0.97; 95% CI, 0.58 to 1.62; *P* = 0.90).

**Table 3:** Time to first observed remission by age group and glomerular disease subtype

GD	Age Group	Hazard Ratio	95% CI	*P* Value
MCD	AYA	REF	REF	REF
	Adult	1.75	0.77 to 3.98	0.18
	Pediatrics	2.18	1.03 to 4.63	0.04
FSGS	AYA	REF	REF	REF
	Adult	0.97	0.58 to 1.62	0.90
	Pediatrics	1.75	0.97 to 3.18	0.07
IgAN	AYA	REF	REF	REF
	Adult	0.58	0.37 to 0.91	0.02
	Pediatrics	1.18	0.68 to 2.05	0.57

Adjusted hazard ratios for time from active disease or relapse to first observed remission comparing pediatric and adult groups to adolescent and young adult (reference). AYA, adolescent and young adult; CI, confidence interval; GD, glomerular disease; IgAN, IgA nephropathy; MCD, minimal change disease; REF, reference.

## Discussion

This study highlights AYA patients with GD as a distinct population with unique clinical features and disease trajectories compared with younger pediatric and older adult patients. AYA with MCD had more relapses than adults but no difference when compared with pediatric participants. AYA with IgAN had more relapses than adults. AYA with FSGS and IgAN exhibited faster eGFR decline than pediatric participants. Pediatric participants with MCD achieved first observed remission faster than AYA. AYA with IgAN achieved first observed remission faster than adults.

For this study, we defined AYA as individuals aged 14–25 years, a developmental stage marked by physiologic, cognitive, and psychosocial changes. Unlike the pediatric age group, who remain largely dependent on caregivers, and adults, who generally manage their own health care independently, AYA patients navigate a transitional period marked by a gradual increase in responsibility for their health. This phase often coincides with shifts in support systems, education, employment, living environments, and health care settings.^2–4,6^ Nevertheless, existing literature often overlook the AYA population by employing rigid age-based classifications—pediatric (<18 years) and adult (18 years and older)—that fail to capture the complexities and unique health care needs inherent to this intermediary group.^25,26^

Our findings indicate that adult patients presented with more advanced kidney disease at enrollment compared with the AYA group, as evidenced by lower eGFR, higher levels of proteinuria, and a greater prevalence of hypertension. Notably, despite more severe baseline disease, adults did not exhibit a significantly greater decline in eGFR over time than the AYA group for any GD subtype. By contrast, AYA had more advanced kidney disease at enrollment compared with pediatric participants with lower eGFR and more proteinuria. Among the patients with FSGS and IgAN, this was further reflected in a more pronounced eGFR decline in the AYA group compared with the pediatric group. In addition, CureGN uses different eGFR equations based on age. The CKD in Children Study under 25 equation, used for patients younger than 25 years, has been shown to underestimate eGFR compared with the CKD Epidemiology Collaboration equation.^27,28^ As a result, the true difference in baseline kidney function between the AYA and adult groups may be even greater than our findings suggest.

Disease duration was similar across all age groups, and given the design of the CureGN study, all participants underwent a kidney biopsy within 5 years before enrollment.^22^ This suggests that many participants were captured relatively early in their disease course, thus age at enrollment serves as a proxy for age of onset of disease, although this may not always be true, especially in the pediatric MCD cohort. Pediatric participants had more active disease at enrollment compared with AYA patients, yet they were also more likely to have achieved complete remission before enrollment. This pattern suggests a more dynamic disease trajectory in pediatric populations. Rates of hypertension were similar between pediatric participants and AYA but less prominent in AYA than in adults. Collectively, these findings highlight that some aspects of GD in AYA reflect pediatric GD, some aspects reflect adult GD, and some aspects are unique to AYA.

One of the most notable findings of this study is the difference in relapse rates and time to first observed remission in MCD. Our findings suggest that the course of MCD follows a spectrum of severity based on age. Adults have lower relapse rates than AYA but do not differ in time to remission. Pediatrics may reach remission faster than AYA but did not show difference in relapse rates compared with AYA. These findings are consistent with prior studies. For example, in a study by Keskar *et al.*, adolescents (ages 13–17) with MCD had higher rates of relapse compared with adults.^29^ Chen *et al.* found that adolescents (ages 13–17) with MCD were less likely to achieve remission compared with pediatric patients ≤12 years.^19^ Despite significant differences in relapse and remission rates, the AYA group did not experience differences in eGFR decline compared with either pediatric participants or adults.

These findings must be interpreted in the setting of the unique characteristics of the CureGN pediatric MCD cohort, which includes more treatment-resistant cases due to the necessity of a kidney biopsy for enrollment. In clinical practice, pediatric patients with idiopathic nephrotic syndrome who present with classic features of MCD and respond to initial corticosteroid therapy generally do not undergo biopsy, resulting in underrepresentation of typical steroid-sensitive cases in the cohort.^19^ In addition, patients who never relapsed were excluded from remission analysis and patients who never reached remission were excluded from relapse analysis, which may have influenced our results and may underestimate the magnitude of the relapse and remission effect seen in our study.

Our findings support that age remains an important factor influencing the clinical course of MCD, which is consistent with existing knowledge of the disease. This may relate to immunologic maturation during adolescence and young adulthood which could affect disease reactivation and treatment responsiveness.^25^ Altogether, pediatric patients tend to have a more dynamic course, whereas adults relapse less often. AYA patients fall in between these two age extremes of the spectrum, underscoring the need for tailored approaches to disease monitoring and management.

Among patients with FSGS, relapse rate differences between AYA and pediatric participants and between AYA and adults were not statistically significant. There was no difference in eGFR decline or time to first observed remission between AYA and adults. Conversely, AYA had more eGFR decline when compared with pediatric participants with FSGS and trended toward a slower time to first observed remission. This may reflect cumulative disease burden from delayed recognition of early disease manifestations of this transitional age group. These finding differ slightly compared with other studies. For example, in another CureGN study, Garrity *et al.* found no significant differences in composite eGFR decline or remission rates in FSGS across childhood onset (5–12 years), adolescent onset (13–19 years), young adult onset (20–29 years), and adult onset (30–39 years) disease, although their age definitions differed from those of our study.^21^ Additional studies have shown that in FSGS, no significant differences in progression to ESKD or 40% eGFR decline have been observed between adolescents (ages 13–17) and younger or older age groups.^18^

In patients with IgAN, relapse rates and time to first observed remission did not differ between AYA when compared with pediatric participants. Despite this, AYA still had faster eGFR decline compared with pediatric participants. Conversely, AYA experienced more relapses but faster time to reach first observed remission when compared with adults. Despite the differences in relapse and remission, eGFR decline over time did not differ significantly between adults and the AYA group. Other studies have shown similar findings. In IgAN, young adults (ages 14–29) have been found to have distinct histopathologic features (more crescents, less sclerosis, and vascular lesions) but similar renal survival compared with older adults.^30^

Historically, management strategies for less common GDs in one age group have often been adapted from the more prevalent age group (*e.g*., pediatric regimens used for adult MCD and IgA vasculitis or adult strategies applied to pediatric MN).^25^ However, adolescents are often managed according to either pediatric or adult guidelines without clear evidence that outcomes or disease courses align. Moreover, clinical trials frequently exclude patients younger than 18 years, resulting in a gap in data for certain AYA patients.^31^ Our findings support the urgent need for age-specific protocols and inclusion of AYAs in therapeutic trials to guide evidence-based care.

There are some limitations of our study. Our cohort includes those who have undergone a kidney biopsy for their GD. For the pediatric group, this tends to be reserved for those with complicated disease resistant to initial treatments and therefore may lack generalizability. Our study likely overestimates the relapse rate and underestimates the remission rate of pediatric patients with suspected MCD. The eGFRs were capped at 140 ml/min per 1.73 m^2^, to account for potential hyperfiltration commonly seen in children with MCD, which may have caused flattening of eGFR decline slope, specifically in this group.^19^ The relatively short follow-up period may have limited the ability to detect longer-term differences in disease progression as well. Our definition of remission in IgAN required absence of hematuria; however, some patients with well-controlled disease may still have microscopic hematuria.^32^ As a result, our definition may not have captured all IgAN patients with well-controlled disease. In addition, it remains uncertain how access to newer therapies, often only approved for those older than 18 years, may have influenced treatment strategies and outcomes in our study.

Another limitation of the study is related to the low retention rate among adolescents, particularly those 14–17 years, who had the lowest retention compared with other age groups in the CureGN database. This finding may reflect broader challenges in retaining this population in clinical care, consistent with existing literature on AYA with chronic disease. This group is known to struggle with treatment adherence and health care disruption.^6,33^

The variability in disease patterns among AYA patients in the different GD subtypes likely reflects a combination of biologic, developmental, and health system-related factors. Further investigation is needed to understand the underlying reasons for outcome differences in AYA patients. In particular, the role of health care transitions, psychosocial factors, and treatment access should be explored. Dedicated research in AYA populations is essential to develop age-specific diagnostic and therapeutic frameworks that are aimed at meeting this group's unique health care needs.

AYA patients with GD represent a distinct group, differing from both pediatric and adult populations for relapse frequency, changes in kidney function and remission patterns. These differences highlight the need to consider AYAs separately when designing care strategies and clinical trials. Rather than a binary distinction between pediatric and adult disease, outcomes may exist along a continuum influenced by age and developmental stage. Recognizing this spectrum and addressing the unique needs of AYA is crucial to improving outcomes and ensuring equitable, evidence-based care across the lifespan.

## Data Availability

Data belong to a third party, and authors are not authorized to share the data. Third Party: CureGN. Reason for Restriction: It contains clinical information from patients that needs to be protected and kept private to comply with regulations. The dataset can only be accessed by approved investigators. The dataset from CureGN can be requested at (https://www.curegn.org/for-researchers#ancillary-studies).

## Declarative Statements

This study includes clinical experimentation and received Institutional Review Board or Ethics Committee approval. All patients provided written informed consent.
